# Functional characterization of *SORL1* variants in cell-based assays to investigate variant pathogenicity

**DOI:** 10.1098/rstb.2022.0377

**Published:** 2024-04-08

**Authors:** Elnaz Fazeli, Elham Fazeli, Petr Fojtík, Henne Holstege, Olav M. Andersen

**Affiliations:** ^1^ Department of Biomedicine, Danish Research Institute of Translational Neuroscience, Aarhus University, Aarhus 8000, Denmark; ^2^ Department of Human Genetics, Amsterdam University Medical Center, Amsterdam Neurosocience, Vrije Universiteit, 1081 HV Amsterdam, The Netherlands

**Keywords:** *SORL1*, SORLA, receptor maturation, pathogenic variants, Alzheimer's disease

## Abstract

SORLA, the protein encoded by the *SORL1* gene, has an important role in recycling cargo proteins to the cell surface. While SORLA loss-of-function variants occur almost exclusively in Alzheimer's disease cases, the majority of *SORL1* variants are missense variants that are individually rare and can have individual mechanisms how they impair SORLA function as well as have individual effect size on disease risk. However, since carriers mostly come from small pedigrees, it is challenging to determine variant penetrance, leaving clinical significance associated with most missense variants unclear. In this article, we present functional approaches to evaluate the pathogenicity of a *SORL1* variant, p.D1105H. First, we generated our mutant receptor by inserting the D1105H variant into the full-length SORLA-WT receptor. Then using western blot analysis we quantified the effect of the mutation on maturation and shedding of the receptor for transfected cells, and finally applied a flow cytometry approach to quantify SORLA expression at the cell surface. The results showed decreased maturation, decreased shedding, and decreased cell surface expression of D1105H compared with wild-type SORLA. We propose how these approaches can be used to functionally assess the pathogenicity of *SORL1* variants in the future.

This article is part of a discussion meeting issue ‘Understanding the endo-lysosomal network in neurodegeneration’.

## 1. Introduction

The *SORL1* gene encodes an endosome sorting receptor, called sortilin-related receptor 1 (abbreviated as SORLA or SORL1), that for neurons is important for recycling of cargo to the cell surface including receptor proteins with vital roles for synaptic activity. However, among the best-understood cargo proteins for SORLA-dependent sorting is the amyloid-precursor protein (APP). It is well established that impaired sorting of APP in the endo-lysosomal system leads to an elevated production of the amyloid β-peptide, which is one of the cardinal pathologies of Alzheimer's disease (AD) [1].

The SORLA protein has multiple *N*-glycosylations sites, and by SDS-PAGE analysis two distinct bands both around 250 kDa in size can be observed: the slowest migrating band corresponds to a form where several *N*-glycans have been modified to the complex-type forms, while the faster migrating form has all its *N*-glycans in the high-mannose form [2]. While the exact mechanism is not yet understood, both forms can be found at the cell surface of neurons [3] and for cells with exogenous SORLA expression, but only the mature form is subjected to cleavage by tumour necrosis factor-α converting enzyme (TACE) and ectodomain shedding [2]. By contrast, the immature form is the only form of the receptor located within the earliest compartments of the secretory pathway, including the endoplasmic reticulum (ER) [2,4]. Importantly, the conversion of immature to mature glycan profile relies on efficient trafficking in the endocytic and endosome recycling pathways and depends on the efficient expression of the retromer sorting complex to which SORLA binds [2,3,5].

The *SORL1* gene has a long history for its association with Alzheimer's disease; this was initiated in 2007 when a candidate gene approach identified several single nucleotide polymorphisms (SNPs) in *SORL1* that were associated with sporadic and late-onset AD [6]. Later, a large number of independent genetic case–control studies confirmed these associations, and with genome-wide association studies (GWAS), several SNPs in *SORL1* reached genome-wide significance for their association with AD [7–10].

More recently, the possibility to perform whole-exome sequencing has resulted in the identification of an overwhelming number of *SORL1* variants in AD patients. While truncating *SORL1* variants are almost exclusively found in AD cases, the large majority of *SORL1* variants are missense variants, each with a possible different mechanism in SORLA function and individual effect size on AD risk, ranging from benign to deleterious ([7,11] and Henne Holstege M. L. *et al.* 2024, in preparation). Most of these variants are very rare and some occur only in a few individuals and their family members, which complicates classical genetic linkage studies of co-segregation. To date, more than 500 different missense *SORL1* variants have been described: while a few of them have been functionally tested for an effect on receptor function [12–14], the effect on SORLA activity has not yet been established for the vast majority of missense variants. One reason for this is that it is still an open question *how* to address pathogenicity for this sorting receptor.

For variants in proteins with enzymatic activity, it is relatively simple to define variant-effects based on altered substrate-based activity assays. The impact of genetic variants in proteins with a specific function, such as endocytosis of specific substrates, can also be determined using function-dependent assays. For example, variants in the *LDLR* gene, encoding the low-density lipoprotein receptor, may decrease receptor activity and thereby affect the concentrations of circulating extracellular cholesterol levels [15,16].

### (a) But how to estimate the effect of a variant in an endosome sorting receptor?

We have recently performed an analysis to predict the pathogenicity of variants in *SORL1* based on pathogenic variants in proteins with homologous domains including *LDLR* [17]. But there is clearly a huge unmet need to also perform a set of functional cell-based assays to allow establishing variant pathogenicity based on experimentally addressed criteria. Here, we present a method to quantify the level of SORLA receptor at the cell surface of transfected cells and discuss how to interpret the expression at the cell surface relative to previously established methods on maturation and shedding. We suggest that the combination of these assays can be a valuable tool to determine the pathogenicity of other *SORL1* variants.

## 2. Methods

### (a) Site-directed mutagenesis

The D1105H variant was inserted in the expression construct for the full-length SORLA-WT receptor (previously described [18]), as well as a C-terminally green fluorescent protein (GFP)-tagged full-length SORLA-WT receptor, using a site-directed mutagenesis kit (Agilent Technologies, QuikChange no. 200521) according to the manufacturers' instructions. The following pair of primers were used: Forward: 5′-CTTTGACAACGACTGTGGACACATGAGCGATGAGAGAAAC-3′, and Reverse: 5′-GTTTCTCTCATCGCTCATGTGTCCACAGTCGTTGTCAAAG-3′.

### (b) Cell transfection and western blotting

Approximately 5 × 10^5^ HEK293 or N2a cells were seeded on six-well plates and transfected with expression constructs for SORLA-WT or SORLA-D1105H, using the Fugene 6 Transfection Reagent kit (Promega) according to the manufacturer's instructions. Cell medium was changed to serum-free conditional medium 24 h post-transfection, cells and medium were harvested after 48 h and cells were lysed using lysis buffer (Tris 20 mM, EDTA 10 mM, Triton-X 1%, NP40 1%). Thirty microlitres of medium samples (from conditions where we seeded equal number of cells and controlled for comparable concentration of the cell lysate) and 20 μg of lysate samples were mixed with NuPAGE LDS sample buffer (Invitrogen, no. 2463558) supplemented with β-mercaptoethanol (Sigma) and separated on SDS-PAGE using 4–12% NuPAGE Bis-Tris gels (Thermo). Proteins were transferred to nitrocellulose membranes (Thermo) and blocked for 1 h at room temperature in blocking buffer (Tris-base 0.25 M, NaCl 2.5 M, skimmed milk powder 2%, Tween-20 2%). Membranes were then incubated overnight at 4°C with LR11 antibody (1 : 1000, BD Biosciences, no. 612633) and β-actin (1 : 2000; Sigma, no. A5441). Next day, the membranes were washed twice each 5 min in washing buffer (CaCl_2_ 0.2 mM, MgCl_2_ 0.1 mM, HEPES 1 mM, NaCl 14 mM, skimmed milk powder 0.2%, Tween-20 0.05%) and were incubated for 1 h at room temperature with horseradish peroxidase (HRP)-conjugated secondary antibody (1 : 1500; Dako, no. P0260). Membranes were then washed five times each 5 min and finally detected with FEMTO detection reagent (Thermo, no. 34095) and visualized by means of an iBright1500 scanner. Quantification was performed by densitometric analysis in ImageJ and data were plotted in GraphPad Prism 9.5.0.

### (c) Flow cytometry

Cell surface level of SORLA was analysed by flow cytometry in live, transfected HEK293 cells. Briefly, HEK293 cells were transiently transfected with either SORLA-WT, SORLA-D1105H, SORLA-WT-GFP or SORLA-D1105H-GFP plasmid. Twenty-four hours post-transfection, cells were collected by mild trypsin treatment (0.25% trypsin, 2.21 mM EDTA, 37°C, 5 min) [19], pelleted and resuspended in phosphate-buffered saline (PBS pH 7.4). Cells were blocked in blocking buffer (PBS pH 7.4, 0.5% BSA) for 15 min and immunostained for 1 h with rabbit anti-sol-SORLA primary antibody (5387, C. M. Pedersen, Aarhus University; [18]) at 4°C, followed by washing two times with PBS pH 7.4 and 30 min incubation with donkey anti-rabbit Alexa-Fluor 647 secondary antibody (ThermoFisher, A31573) in the absence of detergent. Cells were then washed three times and then resuspended in flow buffer (PBS pH 7.4, 2% fetal bovine serum (FBS), 1% glucose) and finally were analysed using a NovoCyte 3000 flow cytometer equipped with three lasers and 13 fluorescence detectors (Agilent, Santa Clara, CA). GFP and Alexa-Fluor 647 fluorophores were excited by the 488 and 640 nm lasers, respectively. Results were analysed using FlowJo v10.8.1 software (BD Life Sciences) and data were plotted in GraphPad Prism 9.5.0.

### (d) Immunocytochemistry and confocal microscopy

Approximately 5 × 10^4^ HEK293 cells were seeded on poly-L-lysine-coated glass coverslips and were then transfected with plasmid constructs expressing SORLA-WT or SORLA-D1105H using the Fugene 6 Transfection Reagent kit (Promega). Twenty-four hours post-transfection, cells were fixed with paraformaldehyde 4% for 10 min at room temperature, followed by a wash with PBS pH 7.4. Coverslips were then washed two times with PBS containing 0.1% Triton-X and blocked in blocking buffer (PBS pH 7.4, FBS 10%) for 30 min at room temperature. Cells were then incubated overnight at 4°C with a mouse monoclonal anti-SORLA (mAb_AG4; Aarhus University) 1 : 100 antibody, together with an antibody against calnexin (Abcam, AB22595) 1 : 300. Next day, coverslips were washed in PBS with Triton-X 0.1% and incubated in Alexa-Fluor secondary antibodies (donkey anti-mouse Alexa-Fluor 568 (ThermoFisher, A10037) and donkey anti-rabbit Alexa-Fluor 488 (ThermoFisher, A21206) for 1 h at room temperature. Coverslips were washed with PBS once and then incubated with Höechst (Abcam, 1 : 50 000) for 10 min at room temperature. The coverslips were then mounted on glass slides using DAKO fluorescence mounting medium (Agilent) and imaging was performed using an Zeiss LSM800 confocal microscope. Images were processed using Zen 3.5 (ZEN lite) software. Colocalization was quantified using the JACOP plugin in ImageJ software and presented as Mander's correlation coefficient. Graphing and statistical analysis of the data were performed with GraphPad Prism 9.5.0.

### (e) Statistical analysis

The data are presented as the mean ± s.d. or mean ± s.e.m. The ‘*N*’ numbers represent the number of biological replicates in each experiment. For the imaging experiment, ‘*n*’ represents the number of cells analysed. Data were analysed using parametric two-tailed Student's *t*-test paired (WB analysis and flow cytometry) or unpaired (immunostaining). Statistical significance was reached with a *p*-value of less than 0.05, indicated as *p* < 0.05 (*), *p* < 0.01 (**) and *p* < 0.001 (***), *p* < 0.0001 (****), or deemed not significantly (n.s.) changed. All statistical analysis was completed using GraphPad Prism 9.5.0 software.

## 3. Results

### (a) The SORLA p.D1105H variant is predicted to be pathogenic

**Figure 1.**
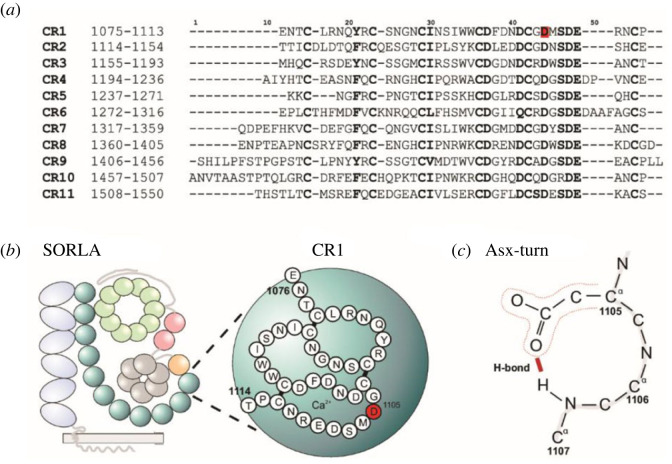
p.D1105H variant characterization. (*a*) Sequence alignment for the 11 CR-domains of SORLA. Conserved residues are shown in bold letters. The location of the p.D1105H variant in the first CR-domain is indicated by red highlighting. (*b*) Schematic representation of all SORLA domains with a close-up on CR1 indicating D1105 in red. (*c*) Schematic representation of Asp-1105 involved in forming a structure known as the ‘Asx-turn’, containing a classical 10-atom ring. The predicted structure represents the sidechains of Asp-1105 (narrow red line), making a hydrogen bond (thick red line) to the backbone of the Ser residue at position 1107.

Here, we focused on the 11:121440955_G > C variant of *SORL1*, leading to the p.D1105H substitution in SORLA, which affects Asp-1105, which is located within the first complement-type repeat (CR)-domain of SORLA at a domain sequence position that is strictly conserved among CR-domain sequences (figure 1*a,b*).

The aspartate residue at this domain position contributes to the folding and stability of CR-domains where its sidechain has a structural role in hydrogen-bonding to the backbone amides of two other residues, including the backbone from the residue at two positions further down the polypeptide chain (i.e. Ser-1107) in a 10-atom ring-structure critical for bending the peptide chain known as the ‘Asx-turn’ [20] (figure 1*c*). The stabilizing role of this structure is further obtained by the sidechain of the conserved serine making an additional hydrogen bond to the backbone of another residue located about 20 residues upstream from the Asx-turn motif [21].

Based on the strong sequence conservation and the structural importance of the aspartate sidechain, we previously applied a domain-mapping of disease mutation (DMDM) approach for known variants of SORLA, to show how variants that affect Asp-1105 are predicted as highly likely to be pathogenic [17]. In short, this approach relies on identifying known disease-mutations at the same domain position in homologous proteins, which will inform whether substitution of residues at a given domain position is tolerated [22]. Using this approach we found strong evidence that variants that affect the aspartate of the Asx-turn in CR-domains from LDLR lead to impaired receptor activity, which in the case of LDLR (ie. variants p.D175N, p.D175Y and p.D224V) results in elevated levels of circulating cholesterol and causes familial hypercholesterolaemia [23–25]. For the homologous LRP4 protein, we also noticed how a mutation that affects the aspartate at the same domain position (i.e. p.D137N of LRP4) is considered causal for Cenani–Lenz syndactyly syndrome [26]. In a subsequent analysis of a previously published case–control study [7], we observed variants p.D1105H (for CR1) and p.D1146N (corresponding to Asx-turn aspartate for CR2) of SORLA in a 64- and a 48-year-old patient whereas no controls had any variant that affected the aspartate of the Asx-turn [27]. In aggregate, these different findings suggest that the *SORL1* p.D1105H is a pathogenic variant for AD.

### (b) Maturation and shedding defects in transfected HEK293 and N2a cells

**Figure 2.**
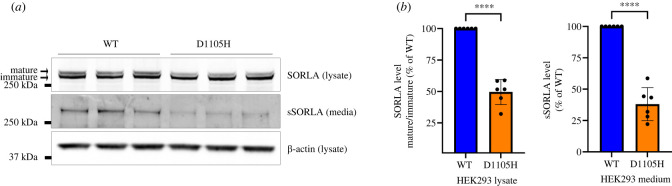
Western blot analysis of SORLA maturation and ectodomain shedding in transfected HEK293 cells. (*a*) Representative western blotting of lysate (SORLA; β-actin) and medium (sSORLA) samples from HEK293 cells transfected with SORLA-WT or SORLA-D1105H. The migration of mature and immature SORLA in the lysate blot is indicated by arrows. (*b*) Densitometric analysis of signals from HEK293 lysate and medium. The signal for D1105H is expressed relative to the wild-type (WT). Results are expressed as mean ± s.d. and analysed by parametric two-tailed paired *t*-test. Significance was defined as a value of *p* < 0.0001 (****). *N* = 6 independent experiments.

We have previously shown how shedding of the ectodomain by TACE is only possible for the SORLA isoform that carries matured *N*-glycans (called mature SORLA) in comparison with the other cellular form of SORLA, which carries a mix of mature and high-mannose *N*-glycans (called immature SORLA), despite both isoforms being present at the cell surface [2]. To determine how p.D1105H affects receptor maturation, we used HEK293 cells, which allow clear discrimination between mature and immature SORLA by western blot analysis of lysates of transfected cells. For HEK293 cells transfected with a wild-type SORLA construct, we observed a clear doublet for western blots, confirming the presence of both immature and mature receptors, whereas lysates from cells expressing p.D1105H showed significantly less signal for the mature SORLA isoform (figure 2*a,b*).

This observed decrease of receptor maturation suggests that the mutated receptor is retained in early compartments of the secretory pathway, as also demonstrated for homologous proteins like LDLR when they carry variants corresponding to mutations that hinder correct folding of the protein domain and thus lead to receptor retention in the ER [28,29].

**Figure 3.**
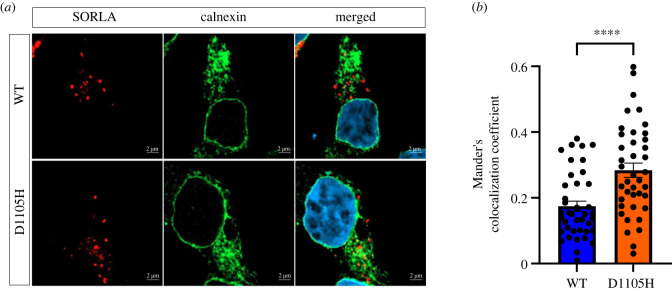
Increased localization of SORLA-D1105H in endoplasmic reticulum (ER). (*a*) HEK293 cells transfected with either wild-type (WT, top row) or D1105H (bottom row) and stained for SORLA (red) and the ER marker calnexin (green). The nuclei were visualized with Höechst (blue). (*b*) Bar graph presents quantifications of colocalization coefficients represented as Mander's colocalization coefficient between calnexin and either WT or D1105H. Data are shown as mean ± s.e.m. and analysed by parametric two-tailed unpaired *t*-test. Significance was defined as a value of *p* < 0.0001 (****). Numbers of cells that were used for imaging were *n* = 41 (WT) and *n* = 40 (D1105H). *N* = 2 independent experiments.

To test whether also SORLA with a mutation within a CR-domain Asx-turn aspartate causes increased expression in the ER, we transiently transfected HEK293 cells with either SORL1-WT or SORL1-D1105H constructs, and analysed intracellular colocalization of both wild-type and mutant receptor with calnexin, which is an ER-resident marker. Using confocal microscopy and determination of the degree of colocalization represented by Mander's correlation coefficient, we found that the localization of p.D1105H is significantly increased in the ER compared with the wild-type protein (WT: 0.17 ± 0.01 (*n* = 41); D1105H: 0.28 ± 0.02 (*n* = 40); *p* < 0.0001) (figure 3*a,b*).

This retention of SORLA in the ER hinders the trafficking of the receptor to the cell surface, from where it can be proteolytically cleaved by TACE—a process that leads to shedding of the entire ectodomain into the cell culture medium [2,30]. Consistent with the increased ER-localization, using western blot analysis of the medium samples from transfected HEK293, we found a significant decrease in the shedding of SORLA-D1105H compared with the wild-type receptor (figure 2*a,b*).

**Figure 4.**
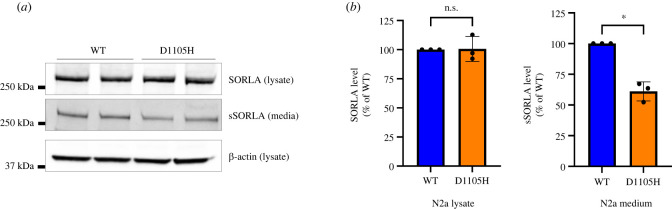
Western blot analysis of SORLA shedding in transfected N2a cells. (*a*) Representative western blotting of lysate (SORLA; β-actin) and medium (sSORLA) samples from N2a cells transfected with SORLA-WT or SORLA-D1105H. (*b*) Densitometric analysis of signals from N2a lysate and medium. The signal for the D1105H is expressed relative to the wild-type (WT). Results are expressed as mean ± s.d. and analysed by parametric two-tailed paired *t*-test. Significance was defined as a value of *p* < 0.05 (*) or not significantly (n.s.) different. *N* = 3 independent experiments.

To confirm this observation we repeated the analysis for shed, soluble SORLA (sSORLA) using transfected N2a cells, with a stronger resemblance to neurons, and found a significantly decreased level of sSORLA in the medium from cells transfected with the D1105H mutant receptor (61 ± 7.8% of wild-type receptor, *p* < 0.05) (figure 4*a,b*). We noticed that for N2a cells it is not possible to obtain a similar clear doublet signal for cell-associated SORLA in lysates tested by western blotting using experimental conditions similar to those for the parallel experiment with HEK293 cells. We quantified the total level of SORLA in N2a lysate (both mature and immature), suggesting similar transfection efficiency of the two constructs (figure 4*b*).

### (c) Cell surface expression measured by flow cytometry

Next, we also tested the cell surface expression for the p.D1105H variant using an independent and quantitative flow-cytometry-based approach. In order to quantify SORLA level at the cell surface relative to the total expression of the protein, thus enabling control for possible differences in transfection efficiencies, we inserted the D1105H variant in an expression construct for SORLA C-terminally fused to the sequence of green fluorescence protein (GFP): SORLA-WT-GFP. HEK293 cells were then transfected with GFP-tagged constructs for either wild-type or D1105H and were analysed by flow cytometry.

**Figure 5.**
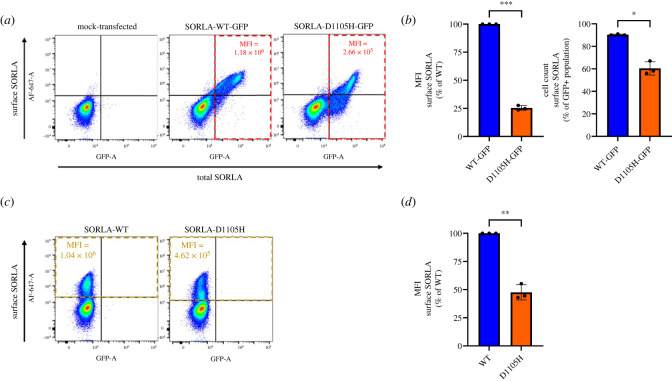
Reduced localization of SORLA-D1105 on the cell surface. (*a*) Flow cytometry dot plot presenting cell surface (Alexa-Fluor 647 (AF-647) fluorescence) and total (GFP fluorescence) SORLA in live single HEK293 cells expressing SORLA-WT-GFP and SORLA-D1105H-GFP. Vertical and horizontal lines show the thresholds for GFP- and Alexa-Fluor 647-positive cells, respectively. The cells inside the red dashed rectangles (total SORLA, GFP+) represent all transfected cells expressing SORLA-GFP, and the numbers show the MFI of AF-647 signal inside the rectangles. (*b*) Bar plot of AF-647 median fluorescence intensity (MFI) signal created from the population of GFP+ on the left, and bar plot of the cell count of surface SORLA to total SORLA (GFP+) on the right. (*c*) Flow cytometry dot plot from HEK293 cells expressing untagged SORLA-WT and SORLA-D1105H. The cells inside the yellow dashed rectangles represent those expressing SORLA at the cell surface (surface SORLA, AF-647+), and the numbers show the MFI of AF-647 signal inside the rectangles. (*d*) Bar plots of AF-647 MFI signal created from the population of surface SORLA. 'mock-transfected' represents the cells that went through the transfection process without addition of any plasmids (treated with transfection reagent only). The signal for the D1105H median intensity is expressed relative to the wild-type (WT). Results are expressed as mean ± s.d. and were analysed by parametric two-tailed paired *t*-test. Significance was defined as a value of *p* < 0.05 (*), *p* < 0.01 (**) or *p* < 0.001 (***). *N* = 3 independent experiments.

By using the GFP-tagged receptor constructs, the GFP signal can be used to select and analyse only cells that have been transfected and thus express SORLA exogenously (total SORLA, GFP+), and then immunostaining of the transfected cells with anti-sol-SORLA primary antibody and an Alexa-Fluor 647 secondary antibody in the absence of any detergent will allow detection of only the cell surface pool of the SORLA protein (surface SORLA, AF-647+) (figure 5*a*). The gating strategy for the flow cytometry analysis is explained in electronic supplementary material, figure S1.

We have presented the quantitative analysis of the data from flow cytometry by two different measurements: first, based on the comparison of the median signal (fluorescence intensity) and second, based on the percentage of the surface SORLA relative to total SORLA (figure 5*b*).

Both these applied methods to compare the cell surface expression level of the receptor showed a significant reduction for the mutated receptor compared with wild-type SORLA at the cell surface, suggesting that both methods can be used to assist in the evaluation of variant pathogenicity.

In order to confirm that the addition of the GFP tag does not interfere with the conclusions of the experiment, we also performed a flow cytometry analysis using HEK293 cells transfected with our untagged SORLA-WT and SORLA-D1105H (figure 5*c*). For this experiment we used untransfected cells that were stained for SORLA at the cell surface for gating to distinguish between signals from endogenous and exogenous SORLA. Also with this experimental setting, we found a significant decrease in the median signal for cell surface expression of the mutant compared with the wild-type (figure 5*d*).

## 4. Discussion

The number of genetic variants for *SORL1* identified in AD patients is continuously expanding, and there is currently no established method allowing a common assessment of the variant-impact on receptor activity. Here we set out to provide functional evidence for impaired SORLA activity for a receptor with the p.D1105H mutation using a number of cell-based assays. Our findings provide functional support for how this variant should be considered as pathogenic, and, moreover, we present and discuss a series of assays that we suggest be employed by any researcher willing to assess variant harmfulness.

### (a) Intrinsic receptor parameters for the assessment of *SORL1* variants using transfected cells

For the SORLA variant p.D1105H, we observed a decrease in mature receptor in lysates from HEK293 cells, decreased shed sSORLA in cell culture medium from experiments with both N2a and HEK293 cells, increased expression in the ER, and a decrease in cell surface localization of the mutated receptor.

Although we observed a correlation between cell surface expression and shedding for the p.D1105H SORLA mutant (both decreased), a similar correlation may not apply to all damaging *SORL1* variants. We have previously shown that both the immature and the mature forms of SORLA reside at the cell surface, while only the mature can be shed [2]. The flow cytometry approach described is unable to distinguish between the two isoforms at the cell surface. Therefore, we cannot exclude that there might be mutant receptors that could lead to an increase of immature SORLA at the cell surface, thus showing a modest decrease for the cell surface but a more severe effect on shedding. Future investigations will inform whether such variants exist and guide us to understand which parameters most accurately capture the mutant effect: the quantification of total SORLA level at the cell surface, or the level of shed (mature) sSORLA in the medium.

Maturation defects have already been described for a number of *SORL1* pathogenic variants displaying reduced level of mature SORLA isoform carrying complex-type *N*-glycosylations [12–14]. It is likely that maturation defects also manifest as a change in the intracellular localization of the receptor. In transfected HEK293 cells, the majority of transfected wild-type SORLA is colocalized with markers of early endosomes (identified by early endosome antigen 1, EEA1) and retromer-coated tubules of the endosome (identified by vacuolar protein sorting-associated protein 35, VPS35), but only a small portion of the receptor localizes in the ER (identified by calnexin) [13]. Accordingly, to assess whether a variant in *SORL1* has a damaging effect on the translated protein, it is also important to study how SORLA is distributed intracellularly using immunocytochemical labelling for SORLA and markers of ER, Golgi and endosomes. For SORLA variants that carry a mutation that leads to receptor misfolding, a larger fraction will be present in the ER, as recently shown for the *SORL1* variant p.R953C [13]. We have also recently shown how the *SORL1* variant p.Y1816C can sort to the endosome, but fails to dimerize, which results in a decrease of retromer-dependent transport to the cell surface [14].

**Figure 6.**
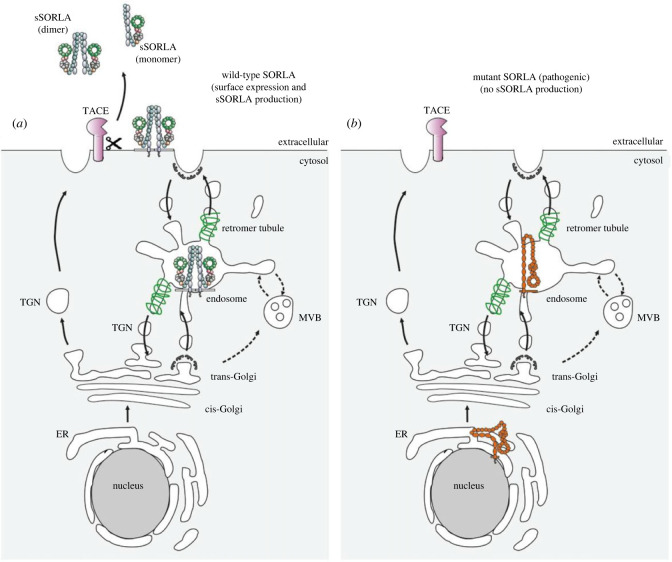
Model of the cellular sorting and shedding of wild-type and mutant SORLA. (*a*) Schematics showing how wild-type SORLA traffics to the endosome, where it can form a dimer and be recycled to the cell surface in retromer-coated tubules. Once at the cell surface in its mature state, it can be shed by tumour necrosis factor-α converting enzyme (TACE) to produce soluble SORLA (sSORLA), which will likely exists in an equilibrium between monomer and dimer form. (*b*) Schematics showing how SORLA with a mutation that leads to folding deficits is mainly retained in the endoplasmic reticulum (ER) (in its immature form) or perhabs can sort to the endosome but in a conformation not compatible to engage in retromer recycling to the cell surface. Both types of mutant SORLA show decreased expression at the cell surface and decreased sSORLA production. TGN: trans-Golgi network; MVB: multi-vesicular bodies.

Accordingly, there are several ways how variants in SORLA can affect cellular localization, but so far it seems a unifying parameter that pathogenic variants will produce less sSORLA (figure 6).

### (b) Cellular and cargo parameters for the assessment of *SORL1* variants using neurons

By today's technologies, it has also become possible to study the effect of mutated SORLA at the endogenous receptor expression level using CRISPR-edited induced pluripotent stem cell (iPSC-derived) neurons. The initial studies using this method focused on the cellular effect of *SORL1 loss-of-function* variants, either using a complete removal of exon 6, corresponding to cells being homozygously deleted for *SORL1* [31] or introducing a mutation corresponding to a frame-shift variant that leads to a truncated SORLA, and where the transcript is likely to be degraded by the non-sense-mediated decay pathway [32]. Such *loss-of-function* alleles of *SORL1* are considered pathogenic. In cells from either of these *SORL1* conditions, the researchers found significantly enlarged endosome structures [32]. It is therefore likely that iPSC neurons with pathogenic *SORL1* missense variants will also have larger endosomes compared with wild-type cells, which suggests that studying endosomal size is an alternative method to determine *SORL1* variant pathogenicity. A recent study has followed this strategy and demonstrated that indeed endosome structures are also increased in cells with different *SORL1* missense variants [33].

It is also possible to study the impact on cargo trafficking in cells with SORLA mutations, as it was shown for cells with the *loss-of-function* variants that both APP and glutamate receptor subunit AMPA1 (GLUA1) were not being efficiently recycled to the cell surface of these cells, and thus, in order to experimentally address if a missense *SORL1* variant leads to a decrease in receptor activity, one could study how these cargo proteins are being sorted to the cell surface [3,34]. Also, a recent study has shown that loss of SORLA in neurons leads to reduced apolipoprotein E (APOE) and clusterin (CLU) levels [35]. Further investigations are needed to assess whether pathologic *SORL1* missense variants can also induce the observed effect or not.

### (c) Patient-derived material for the assessment of *SORL1* pathogenic variants

Instead of introducing the mutation in *SORL1* by CRISPR gene-editing, it is also possible to obtain patient-derived cells to study the impact of SORLA activity. A recent report [36] indeed showed how patient-derived cells from carriers of a *SORL1 loss-of-function* (caused by the frame-shift variant c.4293delC) display similar phenotypes as described above: endosome swelling, defective APP sorting and excessive amyloidogenic processing [37].

Based on the decrease of sSORLA production of pathogenic variants in the medium of transfected cells, it is tempting to speculate that the level of sSORLA is also reduced in the cerebrospinal fluid (CSF) from carriers of pathogenic *SORL1* variants. This is based on the finding that sSORLA in CSF mainly originates from the shedding of neuronal SORLA [38].

**Figure 7.**
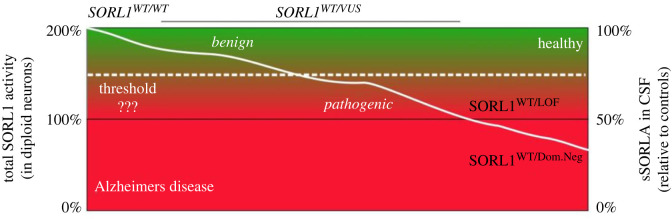
Predicted correlation between *SORL1* activity/sSORLA production and their potential contribution to the development of Alzheimer's disease and assessment of heterozygous carriers of *SORL1* variants of unknown significance (*SORL1*^WT/VUS^). The black horizontal line represents the threshold for *SORL1* activity for developing Alzheimer's disease based on the current knowledge in the field, as defined by haploinsufficient carriers of only one functional allele and one loss-of-function allele (*SORL1*^WT/LOF^). Some variants might behave as dominant-negative, and result in sSORLA cerebrospinal fluid (CSF) levels below 50% owing to decreasing also sSORLA production from the wild-type allele (*SORL1*^WT/Dom.Neg^). The white dashed line is an approximation of the more accurate threshold that needs to be identified by further investigation.

We recently started to address the issue using haploinsufficient minipigs that have only a single functional copy of *SORL1*. We found a 50% reduction in the level of sSORLA in the CSF from heterozygous minipigs compared with wild-type animals [39]. This level of sSORLA in haploinsufficient animals can readily be explained as one *SORL1* allele being expressed at its 100% level and the other allele deleted (a null allele) and producing zero sSORLA. So, in total, of the possible 200% sSORLA, i.e. the level corresponding to two functional alleles present, as in a wild-type animal, the heterozygous minipigs have only half of that CSF sSORLA level. A similar situation is very likely to exist for human carriers of a single copy of a pathogenic *loss-of-function SORL1* variant, we predicted that they would have a 50% reduction of CSF sSORLA levels as compared with individuals with two wild-type *SORL1* copies (figure 7). Indeed we recently analysed sSORLA in CSF from carriers of *SORL1* genetic variants, and observed decreased levels from carriers of *loss-of-function* variants as well as from two carriers of the p.D1105H variant (Henne Holstege M. L. *et al.* 2024, in preparation). Based on our recent identification of how SORLA can form dimers and possible multimers, we speculate that certain pathogenic variants may act as dominant-negative, i.e. the mutant receptor is retained in the ER and is also able to hold back wild-type SORLA in the ER, thus effectively decreasing the amount of sSORLA in the CSF to a level below 50% of normal levels (figure 7).

*But how should we define when a decrease in sSORLA is sufficiently low to correspond to a pathogenic variant that will impair endosome and thus neuronal health?* We suggest that part of the answer will come when we start to correlate the decrease of measured sSORLA in medium from transfected cell lines and of sSORLA in CSF from AD patients. This question can and will only be answered when we start to investigate a larger number of SORLA variants, both less pathogenic and highly pathogenic variants, and begin to correlate the quantified decreases in sSORLA from CSF and the shed fragment from studies in cell cultures. This will not only allow us to identify a threshold value for sSORLA concentrations in the CSF that should indicate if a variant is likely to be pathogenic, but also guide us to how we can use a cell-based assay to obtain a relative value for the decreased sSORLA in medium from cultured cells relative to the amount of shed sSORLA-WT to determine if a variant is pathogenic and where CSF from carriers is not accessible.

For the future, in order to be able to use sSORLA CSF level to assess whether a variant is pathogenic, we will need to understand and identify the ‘normal level’ of CSF sSORLA among carriers of two wild-type *SORL1* copies, which can be used as a reference value for the healthy individuals.

## 5. Conclusion

We have demonstrated that variant p.D1105H leads to impaired maturation of SORLA, which results in less shedding of the ectodomain to the extracellular space, and using a flow-cytometry-based assay we have developed a protocol that can be used to reliably quantify the expression of SORLA at the cell surface of transfected cells. For variant p.D1105H, receptor maturation, shedding and cell surface expression are quantitatively decreased, suggesting that our fluorescence-activated cell sorting (FACS)-based assay can be used to predict pathogenicity of *SORL1* variants using cell-based assays in the future.

In summary, we here propose to use a number of different approaches to functionally assess the impact of any *SORL1* missense variant, as listed in table 1.

**Table 1.** Proposed methods to assess the pathogenicity of *SORL1* missense variants. DMDM, domain-mapping of disease mutation; WB, western blot; ER, endoplasmic reticulum; CRISPR, clustered regularly interspaced short palindromic repeats gene-editing; iPSC, induced pluripotent stem cell; APP, amyloid precursor protein; GLUA1, glutamate receptor subunit AMPA1; SORLA, soluble SORLA.

step	assay	method/tool	outcome (in the case of a pathogenic variant)
1	*in silico*	DMDM approach [17]	located in conserved domain sequence position
2	shedding	WB/reporter activity measurement—medium of transfected N2a	decreased shedding
3	maturation	WB—lysate of transfected HEK293	decreased maturation
4	cell surface expression	flow cytometry—transfected HEK293	decreased cell surface expression
5	receptor localization	immuno-cytochemistry—transfected HEK293	increased ER localization and/or decreased endosome localization
6	endosome swelling	CRISPR-iPSC neurons	increased endosome swelling
7	cargo trafficking	APP and GLUA1 cell-surface surface analysis	decreased cell-surface expression of APP and GLUA1
8	CSF	sSORLA level	decreased sSORLA level

## Data Availability

This article has no additional data.
